# Congenital dermoid cysts of the anterior fontanel

**DOI:** 10.4103/0970-0358.44934

**Published:** 2008

**Authors:** Masood Majed, Farideh Nejat, Mostafa El Khashab

**Affiliations:** Children's Hospital Medical Center, Tehran University of Medical Sciences, Tehran, Iran

Sir,

The congenital dermoid inclusion cyst (CDIC), the most common type of dermoid cyst of the head and neck,[1] is a soft mobile cystic mass covered by normal skin which does not cause any discomfort, pain, or throbbing. There is no communication between the cyst and the intracranial cavity.[2] A dermoid cyst is the most common lesion found around the anterior fontanel.[3] However, CDIC of the anterior fontanel is a rare lesion accounting for 0.2% of all inclusion cysts[4] and 0.1–0.5% of all cranial tumors.[1]

Here we describe eight cases of CDIC of the anterior fontanel, emphasizing the importance of early diagnosis and treatment of these lesions.

**Figure 1:**
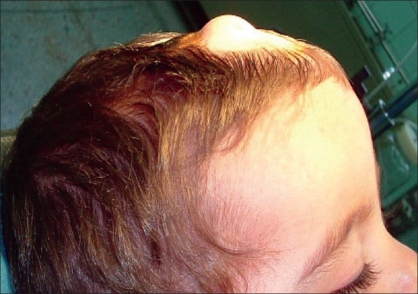
The child with lump around the anterior fontanel

**Figure 2:**
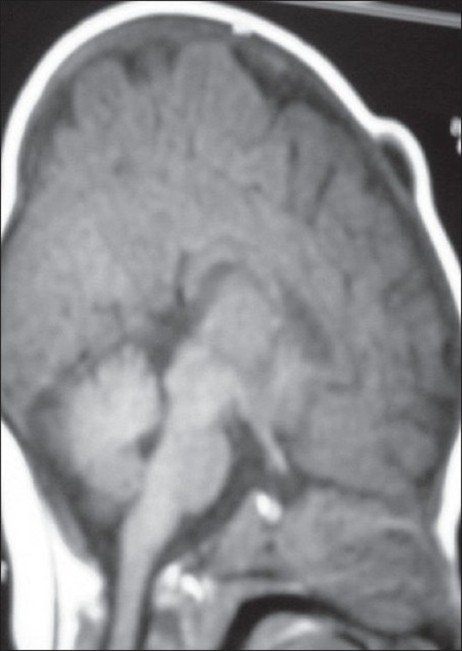
T1-Weighted image, sagittal view of brain MRI of a two month-old infant reveals hypointense mass around the anterior fontanel with intact bone under the lesion

Eight patients referred to the Children's Hospital Medical Centre in Tehran from March 2000 to 2005 for the surgical resection of dermoid cysts around the anterior fontanel. In all cases [Table 1], the lesion was seen at birth as a soft tissue mass around the anterior fontanel, and presented as a firm mobile tumour with progressive growth but no neurological findings. In six cases, the lesion was on the left side. Seven lesions were at the lateral angle, only one was at the anterior angle of the anterior fontanel. The maximum diametres of the lesions varied from 24–54 mm respectively (mean = 36 mm). One patient had an infected dermoid cyst with inflamed, red, and tender skin over the lesion whereas the others had an intact skin covering the lesion [Figure 1]. All patients underwent preoperative neuroimaging: CT scans showed an extracranial heterogeneous hypodense mass and MRI a hypointense lesion on T1-weighted [Figure 2] as well as a hyperintense lesion on T2-weighted images. In four patients, there was a distinct lytic bone lesion under the cyst without any intracranial extension.

**Table 1:** Summary of the clinical, demographic, and neuroimaging characteristics of the studied cases

*Patient no*	*Sex*	*Age*	*Side*	*Site*	*Neuro-imaging*	*Bone defect*	*Skin*
1	Female	2 months	Left	Lateral	MRI	−	Infected
2	Male	4 years	midline	Anterior	MRI	+	Intact
3	Male	4 months	Left	Lateral	CT	−	Intact
4	Male	5 years	Left	Lateral	MRI	+	Intact
5	Female	1 year	Left	Lateral	MRI	+	Intact
6	Female	4 months	Left	Lateral	CT	−	Intact
7	Female	3 months	Left	Lateral	MRI	−	Intact
8	Male	1 year	Right	Lateral	CT	+	Intact

**Figure 3:**
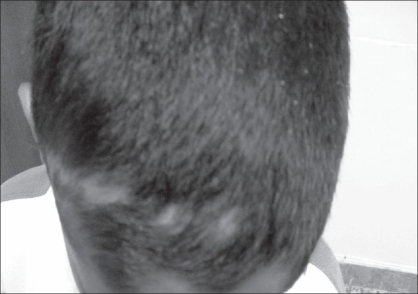
Photography of patient two years after surgery

Patients were operated in the supine position, under sedation and local anaesthesia. The lesion was completely removed through an ellipsoid incision bordering the mass, which was easily dissected from the galea and periosteum, without damage to the sagital sinus or the cyst capsule. Histological investigation showed cysts lined by a thick, stratified squamous epithelium containing skin appendages (hair follicles, sebaceous glands, and sweat glands) in all cases. The postoperative period was uneventful with no recurrence during the follow-up period of 1.5–6 years (mean = 3.2 years) [Figure 3].

Dermoid cyst is a pathologic term for a cyst lined by squamous epithelium containing skin appendages (hair follicles, sebaceous, and sweat glands). It has been classified into three categories: (1) Teratoma type congenital dermoid cysts, (2) Acquired implantation dermoid cysts, (3) CDIC resulting from the inclusion of displaced dermoid cells along the embryonic fusion line.[2] Dermoid cysts of the head and neck are thought to be mostly of the CDIC type, which is usually around the anterior fontanel.[1]

CDIC of the anterior fontanel is described as a slow-growing, nontender, soft lump covered with intact skin. These are usually observed at birth and develop gradually through he accumulation of secretions and internal desquamation.[4]

In spite of the presentation of this cyst at birth, the age of children admitted for surgery in this study ranges from two months to five years. Postponing the operation in some cases can be related to the parents' ignorance or their knowledge of the benign nature of the lesion that permits delayed surgery. Although a female predominance has been reported,[4] we could not find any gender difference. The size of the dermoid cyst has usually been 1–7 cm in the previously reported cases,[4] corresponding to the age of the patient at the time of the diagnosis.[2]

These cysts arise mostly around the anterior angle of the fontanel.[2,5] Interestingly, most lesions (6/8) in this series were at the left lateral angle of the anterior fontanel. This kind of different localization is probably due to the geographic differences.

Most lesions found around the anterior fontanel in infancy are dermoid cysts.[3] Other important pathologies are encephalocoele, meningocoele, sebaceous cyst, lipoma, haemangioma, and cephalohematoma.[1,2,4,6] Precise physical examination and neuroimaging can be useful for differential diagnosis

Computed tomography (CT) scans demonstrate the lesion as an extracranial, encapsulated, low-density mass without enhancement (known as a fluid-containing cyst) that is sometimes accompanied by lytic bone lesions.[2,7] Magnetic Resonance Imaging (MRI) is the most reliable and accurate test[4] and shows the lesion as a low- and high-intensity mass on T1-weighted and T2-weighted images, respectively.[3,7–9] It has been recommended to perform CT or MRI before surgery[9] to determine the extension of the cyst and the presence of a lytic bone lesion.

Although Chaudhari *et al.* found no direct relationship between the depth of the bone defect and time of presentation,[7] all the patients diagnosed during the first year of life (four patients) showed no lytic bone lesion in their imaging in this study. These findings emphasize the importance of an early diagnosis and intervention. Thus, we do not recommend any neuroimaging for mobile cystic masses placed around the anterior frontal in patients younger than a year of age.

Dermoid cysts of the anterior fontanel are excised for cosmetic reasons, to prevent infection, to obtain histological diagnoses, and to rule out malignancy.[3,7–9] The best procedure is a complete resection of the cystic mass with removal of the wall by blunt dissection of the tumour from the underlying tissue (dura or cranium) through an ellipsoid incision bordering the mass.[1] Care should be taken while excising the tumour overlaying an open anterior fontanel or a lytic bone lesion[7] to avoid injury of the underlying structures. Local rather than general anesthesia is preferred in most cases, especially when no lytic bone lesion exists, or the size is not very large.

As seen in our study, there is almost no recurrence if *en bloc* resection of the cyst is performed. Until now, there has been just one case of progression and slow recurrence six years after surgery.[4]
